# A New Role for SAG12 Cysteine Protease in Roots of *Arabidopsis thaliana*

**DOI:** 10.3389/fpls.2018.01998

**Published:** 2019-01-11

**Authors:** Maxence James, Céline Masclaux-Daubresse, Anne Marmagne, Marianne Azzopardi, Philippe Laîné, Didier Goux, Philippe Etienne, Jacques Trouverie

**Affiliations:** ^1^INRA, UNICAEN, UMR 950 EVA, SFR Normandie Végétal (FED4277), Normandie Université, Caen, France; ^2^INRA, CNRS, Institut Jean-Pierre Bourgin, AgroParisTech, Université Paris-Saclay, Versailles, France; ^3^CMABIO3, SF 4206 ICORE, Normandie Université, Caen, France

**Keywords:** cysteine protease activity, N remobilization, reproductive stage, roots, SAG12, N uptake

## Abstract

Senescence associated gene (SAG) 12, which encodes a cysteine protease is considered to be important in nitrogen (N) allocation to *Arabidopsis thaliana* seeds. A decrease in the yield and N content of the seeds was observed in the Arabidopsis SAG12 knockout mutants (*sag12*) relative to the wild type (Col0) under limited nitrogen nutrition. However, leaf senescence was similar in both lines. To test whether SAG12 is involved in N remobilization from organs other than the leaves, we tested whether root N could be used in N mobilization to the seeds. Root architecture, N uptake capacity and 15N partitioning were compared in the wild type and *sag12* under either high nitrogen (HN) or low nitrogen (LN) conditions. No differences in root architecture or root N uptake capacity were observed between the lines under HN or LN. However, under LN conditions, there was an accumulation of ^15^N in the *sag12* roots compared to the wild type with lower allocation of ^15^N to the seeds. This was accompanied by an increase in root N protein contents and a significant decrease in root cysteine protease activity. SAG12 is expressed in the root stele of the plants at the reproductive stage, particularly under conditions of LN nutrition. Taken together, these results suggest a new role for SAG12. This cysteine protease plays a crucial role in root N remobilization that ensures seed filling and sustains yields when nitrogen availability is low.

## Introduction

Many field crop species are high nitrogen (N) demanding plants. In the context of switching to a sustainable agricultural model, a reduction in inorganic nitrogen inputs is required. To reach this goal, it is necessary to deepen the knowledge of the physiological mechanisms related to N management in plants. Among them, senescence metabolism is essential as it allows the redistribution of nutrients from the source organs to the sink organs (Peoples and Dalling, 1988; Masclaux et al., 2000; Gregersen, 2011). In contrast to other elements such as sulfur stored, which is stored as an inorganic form in vacuoles, N is mainly stored as proteins that require some proteolysis steps to generate peptides and amino acids for remobilization to occur during senescence (Hörtensteiner and Feller, 2002; Masclaux-Daubresse et al., 2008; Tegeder and Rentsch, 2010). Such N recycling metabolism is especially important during the reproductive stage to ensure seed N filling. Metabolic events are under the control of a large panel of transcription factors and enzymes (Kusaba et al., 2013) that are also modulated by biotic and abiotic stresses such as N deprivation (Gregersen et al., 2013; Avice and Etienne, 2014; Balazadeh et al., 2014).

Senescence, which corresponds to catabolic pathways occurring before cell death, takes place in all organs (Wojciechowska et al., 2017), but it is commonly accepted that in many plant species, leaves are the main source organs for seed filling (Masclaux-Daubresse et al., 2008). During leaf senescence, the chloroplasts, which contain more than 75% of the total N of the leaf (50% in the form of RuBisCO), are the first organelles to be degraded (Peoples and Dalling, 1988; Hörtensteiner and Feller, 2002). In contrast, the mitochondria and nucleus remain functional until cell death (Lim et al., 2007; Chrobok et al., 2016), to allow the production of energy and the expression of Senescence associated genes (SAGs) that encode proteins necessary for transport and catabolism reactions (Gan and Amasino, 1997).

Protein degradation associated with senescence requires a multitude of proteases (Guo et al., 2004). They belong to five major classes: Cysteine Proteases (CPs), Serine Proteases (SPs), Aspartate Proteases (APs), Metallo Proteases (MPs), and Threonine Proteases (TPs) (Guo et al., 2004). Their specific role in protein breakdown during leaf senescence is not well-known, and the major class overexpressed in many plant species during senescence is CPs (Guo et al., 2004; Poret et al., 2016). The SAG12 papain-like cysteine protease (Noh and Amasino, 1999a,b) is the most strongly induced CP in senescent leaves of *Brassica napus* L. and *Arabidopsis thaliana*, especially in plants cultivated under nitrogen limitation (Desclos et al., 2008; Poret et al., 2016). In addition, high SAG12 protein levels are detected in senescing leaf tissues and in fallen leaves (Desclos-Théveniau et al., 2015). For all these reasons, a major role for SAG12 in N remobilization during senescence has long been proposed. Surprisingly, no difference in the leaf senescence phenotype between Col and KO-SAG12 (*sag12*) plants has ever been observed (Otegui et al., 2005; James et al., 2018). Otegui et al. (2005) showed that a lack of SAG12 did not prevent the formation of senescence associated vacuoles (SAVs), nor was their proteolytic activity affected, suggesting that other cysteine proteases accumulated in SAVs in the senescent leaves of *Arabidopsis thaliana* (Otegui et al., 2005). More recently, James et al. (2018) confirmed the absence of a leaf senescence phenotype in *sag12*. The authors also showed that there was no difference in yield and seed N content between Col and *sag12* when cultivated under optimal N nutrition, but there was lower seed N content and yield in *sag12* when cultivated under limiting nitrogen conditions. The absence of phenotype under high nitrogen was explained by an induction of cysteine and aspartate protease activities in *sag12* that could preserve N remobilization. Nevertheless, the authors did not exclude the potential that better root N uptake in *sag12* could have maintained seed filling and plant productivity. This hypothesis is in agreement with the decrease in seed nitrogen content and yields observed in *sag12* cultivated under LN conditions. Indeed, under this particular N limitation condition, N seed filling can only be achieved by N remobilization, in spite of a possible increase in the N uptake capacity in the roots of *sag12*.

The aim of the present study was to determine whether root N uptake and/or N remobilization are involved in the preservation of seed N filling in *sag12*. Root morphology and N uptake were monitored in Col and *sag12*. Experiments using ^15^N pulse/chase labeling were performed to investigate the distribution of ^15^N and estimate the source and rate of remobilized nitrogen dedicated to seed filling.

## Materials and Methods

### Plant Growth Conditions

*Arabidopsis thaliana* Columbia (Col) and *sag12* (SALK_124030) T-DNA mutants were used in this study. The KO-SAG12 SALK_124030 (*sag12*) was chosen because it was the only germplasm which was available in ABRC stock and recently characterized for some study focused on protease activities (Pružinská et al., 2017; James et al., 2018). Seeds were stratified for 48 h in 0.1% agar (Select agar, Sigma, L’Isle d’Abeau Chesnes, France) at 4°C in the dark and then sown into Eppendorf tubes (0.5 ml) filled with 0.8% agar (w/v) that had their bottoms removed. Plants were placed in a glasshouse on a tank containing 10 L of 3.75 mM ${\mathrm{NO}}_{3}^{-}$ nutrient solution during 44 days. The solutions contained 3.75 mM KNO_3_, 0.5 mM MgSO_4_, 0.25 mM KH_2_PO_4_, 0.2 mM EDTA.NaFe.3H_2_O, 1.25 mM CaCl_2_, 2H_2_O 14 μM H_3_BO_3_, 5 μM MnSO_4_, 3 μM ZnSO_4_, 0.7 μM CuSO_4_, 0.1 μM CoCl_2_, and 0.1 μM Na_2_MoO_4_, and was renewed every week. A batch of plants was cultivated under the same conditions except that a long term ^15^N labeling was performed (5% of atom excess, 3.75 mM K^15^NO_3_ during 44 days) in order to obtain homogeneous ^15^N-labeled plants and to further appreciate the ^15^N partitioning at harvest time (125 DAS). At 44 days after sowing (DAS) all plants were transferred to two contrasting N conditions: High Nitrogen with 3.75 mM N [HN; 1.25 mM Ca(NO_3_)_2_.4H_2_O, 1.25 mM KNO_3_, 0.7 μM (NH_4_)_6_Mo_7_O_24_] and Low Nitrogen with 4.2 μM N [LN; 0 mM Ca(NO_3_)_2_.4H_2_O, 0 mM KNO_3_, 0.7 μM (NH_4_)_6_Mo_7_O_24_]. Photosynthetic photon flux density was 110 mmol m^-2^ s^-1^ and day and night temperatures were 21 and 18°C, respectively. During the first 64 DAS, plants were cultivated with 8 h light/16 h dark photoperiod and then the reproductive stage was induced with a 16 h light/8 h dark photoperiod. Plants were harvested at the vegetative stage (64 DAS) and at the reproductive stage: 85 DAS corresponding to seed filling and 125 DAS corresponding to mature seed stage (Supplementary Figure S1). At each harvest time, the plant compartments present were separated, weighed and stored at -80°C for further analysis. In addition, the root and tip density was performed at 64 DAS with a flat scan (Epson expression 10000XL scanner, Suwa, Japan) coupled with Winrizho software (Regent, Sainte-Anne, QC, Canada).

### Root N Uptake Capacity Analysis

Col and *sag12* plants were cultivated in HN conditions as previously described but without any ^15^N labeling. The root N uptake analysis was performed at vegetative (64 DAS) and reproductive stages (85 DAS) as previously described by (Lainé et al., 1993). Briefly, roots were washed twice for 1 min in a solution of CaSO_4_ (1 mM) before immersion for 5 min in a solution of 250 μM or 2 mM K^15^NO_3_ (99% of atom excess) to study N uptake by the high affinity transport system (HATS) or the low affinity transport system (LATS), respectively. Then roots were rinsed twice in a solution of CaSO_4_ (1 mM) at 4°C for 1 min to stop the N uptake. Roots and shoots were separated and weighed before ^15^N analysis by an isotope mass ratio spectrometer (IRMS, IsoPrime GV Instruments, Manchester, United Kingdom). Uptake capacity was expressed as the total amount of ^15^N in a whole plant per gram of dry roots per hour.

### Isotopic Nitrogen Analysis

N and ^15^N contents were quantified in the different plant compartments with an elemental analyzer (EA3000, EuroVector, Milan, Italy) coupled with an isotope mass ratio spectrometer (IsoPrime IRMS, GV Instruments, Manchester, United Kingdom).

The nitrogen quantity (***NQ***) in the sample was obtained with the following formula: $NQ=\frac{\%N\times MS}{100}$

where MS represents the dry matter of the sample.

The isotopic abundance (***A%***) was determined with the formula: $A\%=100\times \frac{{}^{15}N}{\left({}^{15}N+{}^{14}N\right)}$

With ^15^N and ^14^N representing the amount of ^15^N and ^14^N isotopes, respectively.

The isotopic excess (***E%***) corresponds to the difference between the isotopic abundance of sample (A%) and the N natural abundance (0.3660%): E% = A% - 0.3660%

Finally, the isotopic excess was used to estimate the quantity of ^15^N (μg) (***Q^15^N***): Q^15^N = (E% × QN) × 1000.

### Determination of Amino Acid Content

Ten mg of lyophilized roots were added to 400 μl of MeOH containing 0.625 nmol/μL of norvaline used as internal standard (Sigma, L’Isle d’Abeau Chesnes, France). The mix was stirred for 15 min and then 200 μl of chloroform and 400 μl of ddH_2_O were added. After centrifugation (12000 rpm, 10°C, 5 min), the supernatant was recovered, evaporated and resuspended in 100 μl of ddH_2_O and then filtered on a 0.2 μm membrane before derivatization using an AccQ-Tag Ultra Derivatization Kit (Waters, Guyancourt, France) following the manufacturer’s protocol (Waters, Guyancourt, France). Amino acids were separated and quantified using a UPLC/PDA H-Class system (Waters, Guyancourt, France) with a BEH C18 100 mm × 2.1 mm column.

### Extraction and Quantification of Soluble Proteins

Two hundred mg of frozen root leaf tissue were ground in a mortar with 250 μL of citrate-phosphate buffer (20 mM citrate, 160 mM phosphate, pH 6.8 containing 50 mg of PVPP). After centrifugation (1 h, 12,000 *g*, 4°C), the concentration of the soluble protein extract was determined in the supernatant by protein staining (Bradford, 1976) using bovine serum albumin (BSA) as standard.

### Western Blot of SAG12 Protein

Twenty μg of soluble proteins were denatured by heating at 90°C for 10 min in 4x Laemmli sample buffer with β-mercaptoethanol (Laemmli, 1970). Proteins were separated on an SDS-PAGE Stainfree precast gel (4–15% acrylamide gradient; Mini-PROTEAN^®^, Bio-Rad, Marnes-la-Coquette, France) and transferred onto a polyvinylidene difluoride (PVDF) membrane as previously described by Desclos et al. (2008). The PVDF membrane was incubated overnight in Tris buffer saline – Tween 20 [TBST; Tris 10 mM, NaCl 150 mM, pH 8, Tween 20 0.15% (v/v)] with 3% (v/v) skimmed milk to avoid non-specific hybridization. Immunodetection of SAG12 was performed using an anti-SAG12 specific polyclonal antibody from rabbit provided by Agrisera^®^ (AS14 2771; 1/2000 in TBST) as primary antibody and a second antibody coupled with peroxidase (1/10000 diluted in TBST, Bio-Rad^®^). The quantification of SAG12 was performed by the measurement of the chemiluminescence revealed with an ECL Kit (Bio-Rad^®^, Marnes-la-Coquette, France) using a ProXPRESS 2D proteomic imaging system (PerkinElmer, Courtaboeuf, France).

### Proteolytic Activities

Proteolytic activities of cysteine proteases were determined by *in vitro* protein degradation analysis as previously described in James et al. (2018). Twenty μg of soluble proteins were incubated in a 200 μL reaction volume containing Na-acetate buffer (50 mM, pH 5.5) and 10 μg of BSA (exogenous protein used as loading control). Cysteine protease activities (CP_act_) were obtained by the addition of 50 μM of E-64, a cysteine protease inhibitor dissolved in dimethylsufoxide (DMSO). Furthermore, 2 mM dithiothreitol (DTT) was added to this mixture and total protease activity (TP_act_) was obtained by substituting inhibitors with an equal volume of DMSO. Then, proteins were precipitated with 1 mL of ice-cold acetone either immediately (t_0_), or after incubation for 300 min (t_300_) at 37°C under gentle agitation. After centrifugation (15 min, 16,000 *g*, 4°C), the pellet was dissolved in 2X SDS-PAGE gel loading buffer (140 mM sodium dodecyl sulfate, 200 mM Tris, 20% glycerol, 5% β-mercaptoethanol, 0.3 mM Bromophenol Blue) and heated at 90°C for 10 min. Then the soluble protein extracts were separated on a 4–15% gradient in SDS-PAGE Stainfree precast gels (Mini-PROTEAN^®^ TGXTM Stain Free, Bio-Rad, Marnes-la-Coquette, France) and scanned under UV light with a Gel Doc^TM^ EZ scanner (Bio-Rad, Marnes-la-Coquette, France). The proteolytic activities were quantified by monitoring degradation of four bands (95, 76, 50, and 37 kDa) corresponding to endogenous proteins (EP) as targets of proteolysis at pH 5.5. Proteolytic activities are calculated as follows:

(1) Total protease activity (TP_act_ expressed in %):

$${\mathrm{TP}}_{\mathrm{act}}=\frac{Q_{(EP)to}-Q_{(EP)t300}}{Q_{(tot)to}}\times 100$$

(2) Cysteine protease activity (CP_act_ expressed in %):

$${\mathrm{CP}}_{\mathrm{act}}=\frac{\left[Q_{(EP)to}-Q_{(EP)t300}]-[Q_{(EP)to}-Q_{(EP)t300Inhib.}\right]}{Q_{(tot)to}}\times 100$$

where the amount of EP (Q_(EP)_) at t_0_ and t_300_, with (Inhib.) or without inhibitor, as well as the total amount of soluble proteins (Q_tot_) at t_0_ were quantified by using ImageLabTM software (Bio-Rad, Marnes-la-Coquette, France).

### Extraction and Quantification of RNAs, Reverse Transcription, and PCR Analysis

Total RNAs were extracted from 200 mg of frozen leaf tissue previously ground in a mortar containing liquid nitrogen. The powder was suspended in 750 μL of extraction buffer (100 mM LiCl, 100 mM TRIS, 10 mM EDTA, 1% SDS (w/v), pH 8) and 750 μL of hot phenol (80°C, pH 4). After vortexing for 40 s and after addition of 750 μl of chloroform:isoamylalcohol (24/1, v/v), the homogenate was centrifuged (15,000 *g*, 5 min, 4°C). The supernatant was added to 750 μl of 4 M LiCl solution (w/v) and incubated overnight at 4°C. After centrifugation at 15,000 *g* for 20 min at 4°C, the pellet containing total RNAs was resuspended with 100 μL of sterile water. Then, total RNAs were purified with an RNeasy Mini Kit according to the manufacturer’s protocol (Qiagen, Courtaboeuf, France). Quantification of total RNA was performed by spectrophotometry at 260 nm (BioPhotometer, Eppendorf, Le Pecq, France) before reverse transcription (RT). For RT, 1 μg of total RNAs was converted to cDNA with an iScript cDNA synthesis kit according to the manufacturer’s protocol (Bio-Rad, Marnes-la-Coquette, France) before polymerase chain reaction (PCR) and quantitative PCR (qPCR) analyses.

*SAG12* and 18S rRNA gene expressions were monitored by PCR using 1 μL of cDNA added to 10 μl of a PCR mix containing 250 μM dNTPs, 0.65 μM of forward and reverse primers and 0.5 μM (5 U μL^-1^) Qbiogene Taq polymerase (MP Biomedicals, Illkirch-Graffenstaden, France). The primers were designed with primer3+ software. *SAG12 (At5g45890)*: forward: 5′-GGCAGTGGCACACCAMCCGGTTAG-3′; reverse 5′-AGAAGCMTTCATGGCAAGACCAC-3′ and *18S rRNA (NR_141642):* forward: 5′-CGGATAACCGTAGTAATTCTAG-3′; reverse: 5′-GTACTCATTCCAATTACCAGAC-3′. PCRs were performed in a thermocycler (Applied Biosystems, Courtaboeuf, France) using the following program: 1 cycle at 95°C for 5 min, 25 and 18 cycles for Sag12 and *18S rRNA* including a denaturing step at 95°C for 30 s, a primer hybridization step at 58°C for 45 s and an amplification step at 72°C for 1 min. Each PCR reaction was finished with one cycle at 72°C for 10 min. The identity of each amplicon was checked by sequencing and BLAST analysis. PCR products were separated by electrophoresis on agarose gels (1.2% in TAE 1X with 5 μg mL^-1^ of ethidium bromide) and revealed by illumination with UV light using a Gel-Doc ^TM^ EZ Scanner (Bio-Rad, Marnes-la-Coquette, France).

Additionally, *SAG12* gene expression was monitored using RT-qPCRs analysis with 4 μl of 100× diluted RT product, 500 nM of forward and reverse primers and 1X SYBR Green PCR Master Mix (Bio-Rad, Marnes-la-Coquette, France) in a real-time thermocycler (CFX96 Real Time System, Bio-Rad, Marnes-la-Coquette, France). The program was: 95°C for 3 min followed by 40 cycles (95°C for 15 s followed by 40 s at 60°C). PCR amplifications were performed using specific primers for each housekeeping gene (*EF1-α* : forward: 5′-GCCTGGTATGGTTGTGACCT-3′; reverse: 5′-GAAGTTAGCAGCACCCTTGG-3′; *18S rRNA* : forward: 5′-CGGATAACCGTAGTAATTCTAG-3′; reverse 5′-GTACTCATTCCAATTACCAGAC-3′) and target gene *SAG12* (Forward: 5′-AAAGGAGCTGTGACCCCTATCAA-3′; reverse: 5′-CCAACAACATCCGCAGCTG-3′) as described by Kim et al. (2018). For each sample, the subsequent RT-qPCRs were performed in triplicate. The expression of the target gene in each sample was compared to the control sample (Col at the vegetative stage for each N condition) and was calculated with the delta delta Ct (ΔΔCt) method (Livak and Schmittgen, 2001).

### Transgenic promoterSAG12::UIDA Construct

The SAG12 promoter (*promoterSAG12*), containing the 1–2180 bp sequence found in GenBank under the Accession No. U37336, was cloned from the pSG499 plasmid (Gan and Amasino, 1995) kindly provided by Pr. R. Amasino (University of Wisconsin-Madison, Madison, WI, United States) into the pMDC32 vector between the PmeI and AscI restriction enzyme sites, in place of the 35S promoter, giving the pMAZ01 plasmid. The pMAZ01 plasmid was fully sequenced from the RB to the Nos terminator to verify the absence of any modification especially in the SAG12 sequence. The full-length coding sequence of *UIDA* (GUS-coding gene; from 86 to 1897 of the AJ298139 accession number) was then cloned successively in the pENTR^TM^ vector (Invitrogen, Carlsbad, CA, United States), then into the pMAZ01 vector by Gateway recombination giving rise to the pMAZ02 vector containing the *promoterSAG12*::*UIDA* fusion. The correct GUS sequence was verified by sequencing.

Transgenic Arabidopsis plants carrying the *SAG12* promoter fused to the *UIDA* reporter gene were obtained by floral dipping (Clough and Bent, 1998) and eight homozygous lines of several primary transformants were selected on the basis of their hygromycin resistance and single insertion segregation rate.

### GUS Staining and Observations

Based on the method of Jefferson et al. (1987), tissues from two independent *promoterSAG12::UIDA* homozygous lines were stained overnight at 37°C in 50 mM Na_3_PO_4_ pH 7.0, 5 mM ferricyanide, 5 mM ferrocyanide, 0.05% Triton X-100 and 5-bromo-4-chloro-3-indolyl glucuronide (1 mg mL^-1^). Then after destaining by successive incubations in 50, 75, and 96% (v/v) ethanol, samples were kept in glycerol at 4°C before observation.

The tissues were included in low melting point agarose (5%; w/v) and cut with a vibratome (Microm 650v; Thermo Scientific; United States) before observation with a light microscope (AX70 Olympus, and Olympus SC30 camera, Japan) with the help of cellSens software.

### Statistical Analysis

For all parameters, at least three biological repeats were measured (*n* ≥ 3). All the data are presented as the mean ± standard error (SE). To compare Col with *sag12* data, Student’s *t*-tests were performed after verifying compliance of normality with R software. Statistical significance was postulated at *p* ≤ 0.05.

## Results

### Root Architecture and N Uptake Capacity Are Not Affected in sag12

**FIGURE 1:**
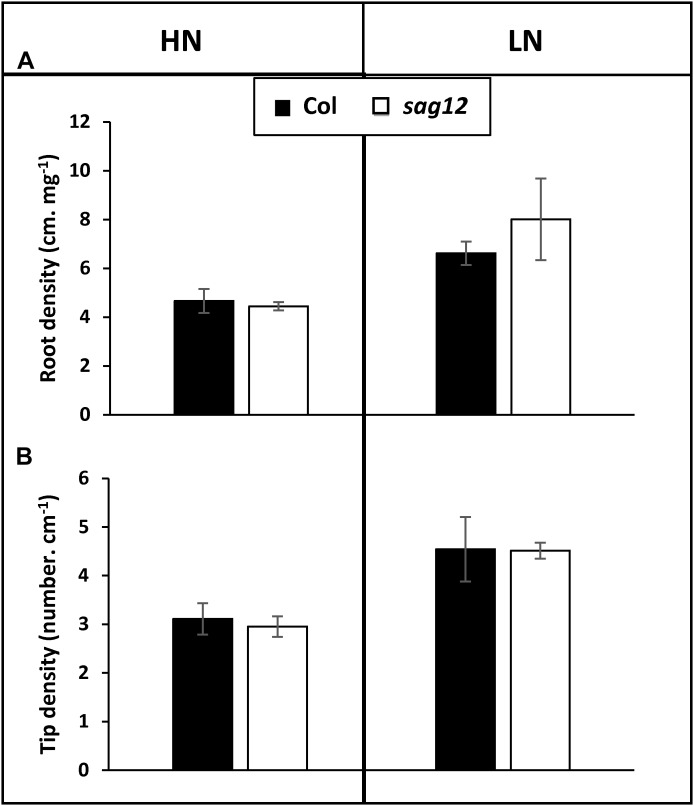
Root architecture is similar in Col and *sag12*. Root density **(A)** and tip density **(B)** of Col (black bars) and *sag12* (white bars) plants cultivated under HN and LN conditions were determined at the vegetative stage (64 DAS). Results are presented as means ± SE (*n* = 3).

**FIGURE 2:**
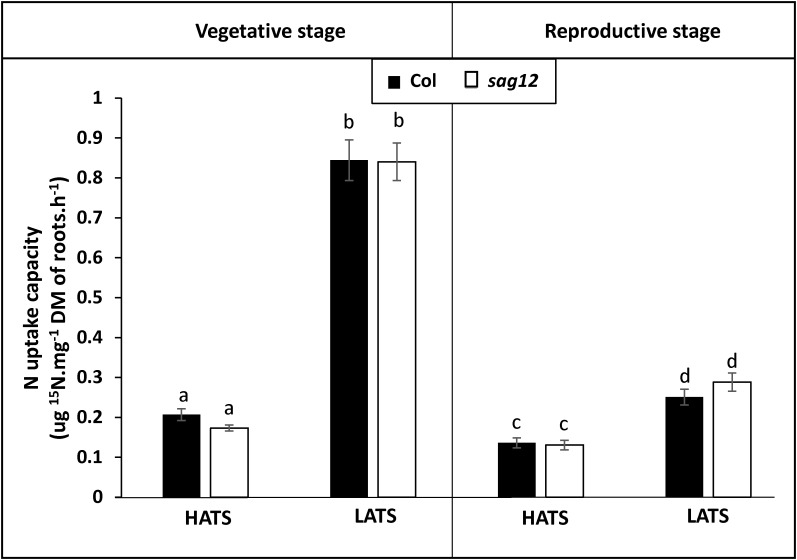
Nitrogen uptake capacities by HATS and LATS are similar in Col and *sag12.* Col (black bars) and *sag12* (white bars) plants were harvested at the vegetative (*n* = 12) and reproductive stages (*n* = 8). N uptake capacities by high (HATS) and low (LATS) affinity transport systems are presented as means ± SE. Different letters indicate statistically significant differences according to Student’s *t*-test (*p* ≤ 0.05).

Irrespective of the N conditions (HN or LN), no significant differences were observed between Col and *sag12* for root (Figure 1A) and root tip (Figure 1B) densities. N uptake capacity was monitored by measuring the amount of ^15^N absorbed per h and mg of root DW. In both the vegetative and reproductive stages, HATS and LATS N-uptake capacities were not different between the genotypes (Figure 2). LATS uptake was higher than HATS in both the vegetative and reproductive stages. Moreover, it could be noted that the N-uptake capacity related to LATS but even more to HATS was significantly lower in the reproductive than the vegetative stage.

### The SAG12 Defect Affects N Allocation Under LN Conditions

**FIGURE 3:**
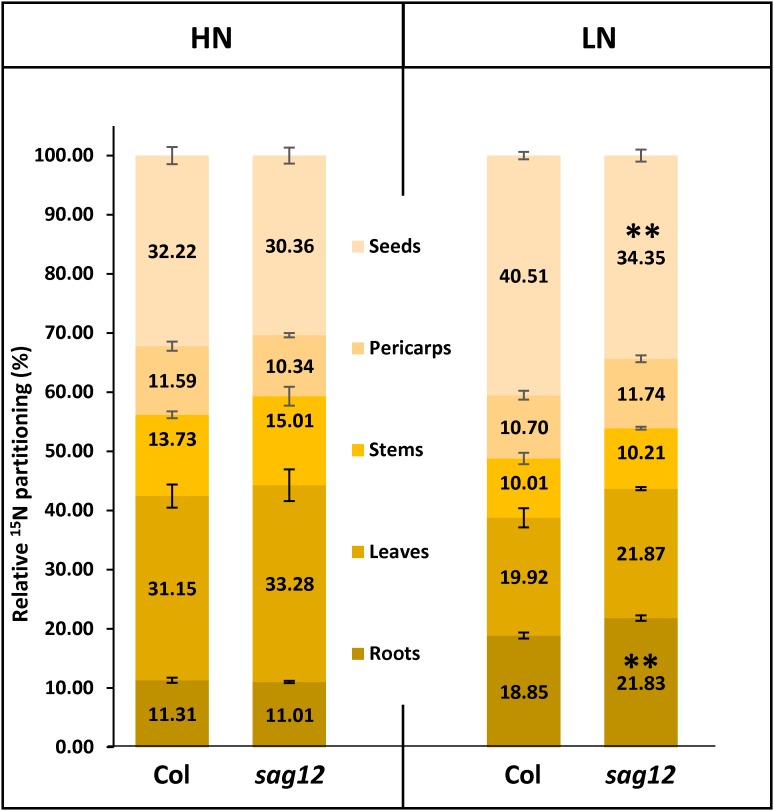
N allocation in roots and seeds is affected in *sag12* under LN conditions. Partitioning of ^15^N in the different plant compartments (roots, leaves, stems, pericarps, and seeds) was calculated from data obtained from plants harvested at seed maturity (125 DAS). Results are presented as means ± SE (*n* = 4). Significant differences between Col and *sag12* are indicated by (^∗∗^*p* ≤ 0.01; *n* = 4).

The plants were labeled with ^15^N for 44 DAS and then grown without ^15^N until harvest at 125 DAS. The similar total ^15^N amounts found in Col and *sag12* at the end of the ^15^N labeling (not shown), confirmed the absence of differences between the genotypes in LATS and HATS uptake capacities at the vegetative stage. Plants were dissected into five compartments (roots, leaves, stems, pericarps, and seeds). Under HN conditions, ^15^N was mainly found in the leaves (Col: 31.15 ± 1.96% and *sag12*: 33.28 ± 2.68%) and in the seeds (Col: 32.22 ± 1.46% and *sag12*: 30.26 ± 1.34%) of the two genotypes (Figure 3) and the partitioning of ^15^N was not significantly different between Col and *sag12*. Under LN conditions, around 78% of the total ^15^N of the plant was distributed in the roots, leaves, and seeds in both genotypes. However, the ^15^N partitioning was significantly different in the seeds and roots of *sag12* and Col. Partitioning of ^15^N in the seeds of *sag12* (34.35 ± 1.02%) was decreased by 6.16% compared to Col (40.51 ± 0.64%) and partitioning of ^15^N in the roots of *sag12* (21.83 ± 0.45%) was conversely increased by 2.97% compared to Col (18.85 ± 0.50%). This then suggests a defect in ^15^N remobilization from the roots to the seeds in *sag12*.

### Protein Content Is Higher in Roots of sag12 Cultivated Under LN Condition

**FIGURE 4:**
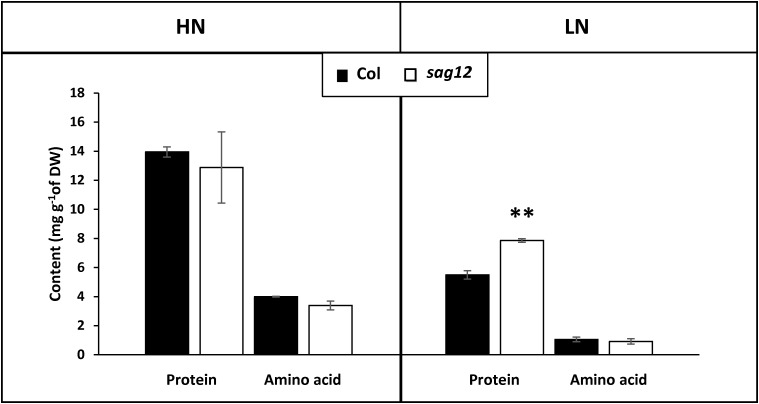
Protein content is higher in roots of *sag12* at the reproductive stage. The roots of Col (black bars) and *sag12* (white bars) plants cultivated under high (HN) and low (LN) nitrogen conditions were harvested at the reproductive stage (85 DAS). Values are means ± SE, *n* = 4. Significant difference between Col and *sag12* (^∗∗^*p* ≤ 0.01; *n* = 4).

As expected, protein and amino acid contents were higher in both genotypes when cultivated under HN compared to LN conditions (Figure 4). Protein content in the roots of *sag12* and Col were similar (around 13 mg g^-1^ of DW) when plants were cultivated under HN conditions. Under LN conditions, the protein concentration was higher in the roots of *sag12* (7.87 ± 0.12 mg g^-1^ DW) than in Col (5.49 ± 0.28 mg g^-1^ DW). Interestingly irrespective of the N conditions, the amino acid contents were not significantly different between the two genotypes (Figure 4).

### SAG12 Is Expressed in Roots at the Reproductive Stage and the Expression Is Higher Under Low Nitrogen Conditions

**FIGURE 5:**
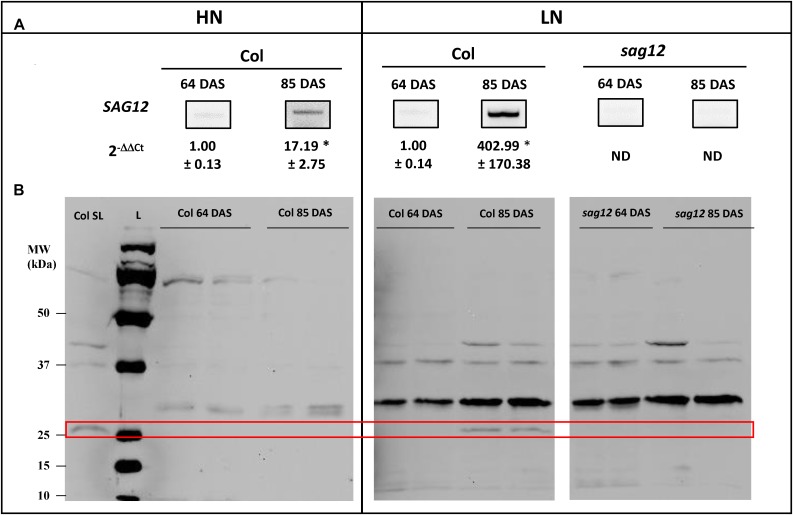
SAG12 is expressed in roots at reproductive stage and especially under LN condition. **(A)** *SAG12* gene expression was monitored using RT-PCR and quantified using RT-qPCR for each condition (HN and LN), the expression of the *SAG12* gene at the reproductive stage (85 DAS) has been relativized with respect to the level of the *SAG12* expression in Col for the corresponding N treatment at the vegetative stage. ND, not detected. For a given nitrogen condition (HN and LN), significant difference between Col at vegetative and reproductive stage (^∗^*p* ≤ 0.05; *n* = 3). **(B)** Detection of SAG12 by Western blot using an anti-SAG12 specific polyclonal antibody provided by Agrisera^®^ (AS142771). The red frame highlight the location of SAG12 protein. The mRNAs and soluble proteins were extracted from roots of Col and *sag12* cultivated under HN or LN conditions and harvested at vegetative (64 DAS) and reproductive (85 DAS) stages. Soluble proteins extracted from senescing leaves of Col (Col LS) were used as positive control for SAG12 expression. The molecular weights (MW; kDa) of the Precision Plus Protein Dual color Standards ladder (Bio-Rad) were indicated.

Low SAG12 transcripts (Figure 5A) and no protein (Figure 5B) could be detected using RT-qPCR and Western blots in the roots of Col at the vegetative stage under low and high nitrogen conditions. In contrast, high SAG12 transcripts levels (Figure 5A) were detected in roots of Col at the reproductive stage and especially under low nitrogen conditions. Accordingly, SAG12 protein was also detected in the roots of Col under LN conditions (Figure 5B). As a control, we checked that SAG12 protein and transcripts were undetectable in the roots of sag12 especially when SAG12 was detected in Col (i.e., reproductive stage and LN).

### SAG12 Is Expressed in the Root Stele

**FIGURE 6:**
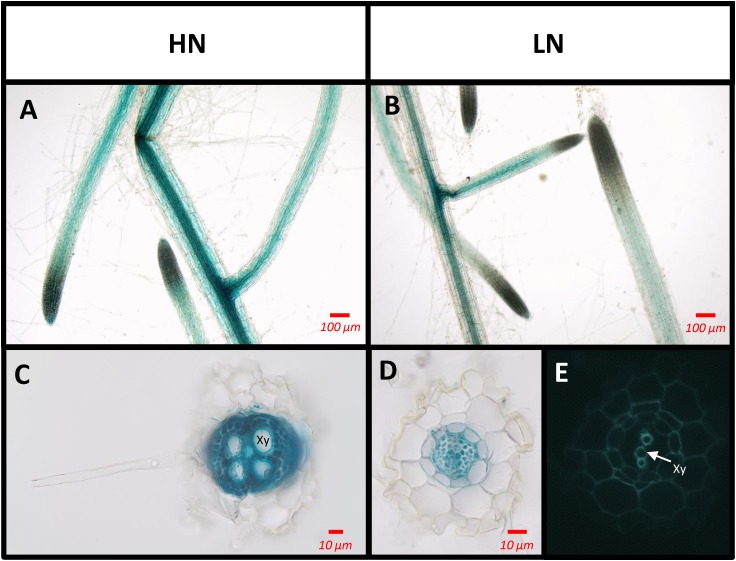
*SAG12* is expressed in the root vascular tissues. The tissues where the SAG12 promoter was active were identified using the *promoterSAG12::UIDA* lines and GUS staining. Roots were observed using a light microscope. Transgenic lines were cultivated under HN **(A,C)** and LN **(B,D,E)** conditions and harvested at the reproductive stage. Representative pictures of the results obtained for whole root **(A,B)** and transverse root sections **(C,D)** are shown. Root section under UV excitation **(E)**. Xy, xylem tissue.

In order to determine the location of SAG12 expression in the root tissues, GUS staining was performed on Arabidopsis plants transformed by the *promoterSAG12::UIDA* reporter fusion. Staining was performed at the reproductive stage, in which *SAG12* genewas previously detected, on plants cultivated under low and high N conditions. Irrespective of the N conditions, GUS staining was observed along the entire length of the root but it was located exclusively in the stele (Figure 6). Surprisingly, although the *SAG12* expression level was higher in the roots of LN cultivated plants (Figure 5), we observed that GUS staining was lower in the LN cultivated roots than in the roots of plants cultivated under HN.

### Cysteine Protease Activity Is Lower in the *sag12* Root Under LN Conditions

**FIGURE 7:**
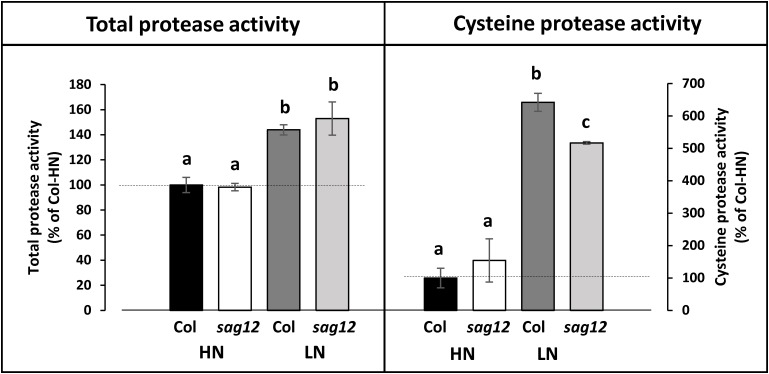
Cysteine protease activity is decreased in roots of *sag12* under LN conditions. Soluble proteins were extracted from roots harvested at the reproductive stage (85 DAS) from Col and *sag12* plants cultivated under high (HN) or low (LN) nitrogen conditions. The cysteine and total protease activities were determined by monitoring the degradation of four endogenous proteins (95, 76, 50, 37 kDa) in the presence or the absence of the cysteine protease inhibitor E64. The protease activities are expressed as a percentage relative to the activity in the Col roots under HN conditions (100%; dot line). Values are means ± SE, *n* = 3. For a given protease activity (total or cysteine), significant differences (*p* ≤ 0.05, *n* = 3) are indicated by different lower case letters.

The total protease activity measured at the reproductive stage (at the optimum pH for SAG12 activity: pH 5.5) did not reveal any difference between Col and *sag12*, regardless of the N conditions (Figure 7). Similar to the total protease activity, a strong increase in cysteine protease activity was observed when plants were cultivated under LN in comparison to HN conditions (Figure 7). While no difference in cysteine protease activity was observed between genotypes when plants were cultivated under HN conditions, the defect in SAG12 led to a significant decrease in cysteine protease activity relative to Col when plants were cultivated under LN conditions (642.11 ± 28.04 vs. 516.88 ± 3.99% in Col and *sag12*, respectively; Figure 7). Such a discrepancy could be attributed to the lack of SAG12 activity.

## Discussion

In a recent study, James et al. (2018) have demonstrated that the absence of SAG12 in plants leads to a decrease in the production of seeds and to a lower N content in Arabidopsis seeds when cultivated under LN conditions. The absence of such a phenotype under HN conditions was explained by the increase in cysteine and aspartate protease activities, which may compensate for the SAG12 defect and sustain N remobilization during seed filling (James et al., 2018). However, although N remobilization is often considered as the major process providing N to the seeds (Tegeder and Masclaux-Daubresse, 2017), the assumption that a better N uptake in *sag12* could supplement the N allocation to the seeds, especially under low N, could not be excluded. Indeed, both ${\mathrm{NO}}_{3}^{-}$ uptake and root architecture are known to be regulated depending on the internal N status of the plant and stimulated under LN conditions (Crawford and Forde, 2002; Nacry et al., 2013; Bellegarde et al., 2017; Gent and Forde, 2017). We here show that whatever the N conditions (LN and HN), Col and *sag12* have the same root architecture (Figure 1). In addition, no matter which developmental stage was investigated (vegetative or reproductive), the N uptake by high (HATS) and low (LATS) affinity transporters was similar in Col and *sag12* (Figure 2). Taken together, these results invalidated the hypotheses that an increase in N uptake (i) supplemented N allocation to the seeds in the *sag12* plants cultivated under HN conditions or that (ii) N content was lower in the seeds of *sag12* compared to Col when cultivated under LN condition (James et al., 2018). In order to monitor the N remobilization for seed filling, at the final stage of plant development (mature seed 125 DAS) we analyzed the distribution of the ^15^N provided in a pulse/chase experiment to Col and *sag12* plants cultivated under HN and LN conditions. Under HN, a similar ^15^N distribution was observed in Col and *sag12* (Figure 3), thus suggesting that in *sag12* (SALK_124030), SAG12 depletion did not alter N remobilisation for seed filling when nitrogen was available. These results are in good agreement with the report of James et al. (2018) showing that the cysteine and aspartate proteases increased in *sag12* might support N remobilization during seed filling. Interestingly, growing plants under low N conditions indicated that the proportion of ^15^N was significantly lower in the seeds of *sag12* in comparison to Col and conversely, significantly higher in the roots of *sag12* compared to the roots of Col (Figure 3). No significant difference between Col and *sag12* was found for ^15^N partitioning in any organs other than the roots and seeds. This then revealed a defect in N remobilization from the roots to the seeds in *sag12* and strongly suggested that the low N content previously observed by James et al. (2018) in the seeds of *sag12* under LN condition was mainly due to the sequestration of N in its roots. Although N remobilization from the root to the seeds is poorly documented, a previous study performed in *Brassica napus* by Rossato et al (2001) showed that more than 11% of the N in seeds came from the remobilization of root N. Moreover, Girondé et al. (2015a) showed that *Brassica napus* genotypes with higher nitrogen remobilization efficiency had a higher contribution of N remobilised from the roots to seeds. Likewise, we observed that the protein concentration was higher in the roots of *sag12* compared to Col, when cultivated under LN, while the amino acid concentrations were unchanged (Figure 4). This work performed in *sag12* (SALK_124030) emphasized the role of SAG12 protease in the proteolysis of root proteins that could serve as a nitrogen source for remobilization under low nitrogen conditions.

From this finding, we then decided to investigate whether SAG12 could be expressed in the root tissue, at least under LN conditions, which has never been described before. Indeed, although the expression of SAG12 in leaves during natural and induced senescence was clearly demonstrated by numerous studies performed in various plant species (Lohman et al., 1994; Gan and Amasino, 1997; Desclos et al., 2009; Parrott et al., 2010; Carrión et al., 2013; Singh et al., 2013; Poret et al., 2016; Curci et al., 2017), to our knowledge there was no evidence for SAG12 expression in plant root tissue. In the present work the SAG12 transcripts and proteins were detected in the roots of *Arabidopsis thaliana* especially at the reproductive stage and were much higher in roots of plants cultivated under LN than under HN conditions (Figure 5). This makes sense because sink strength is known to be stronger at the reproductive stage due to the maturation of seeds and also stronger under LN conditions. The GUS staining of *promoterSAG12::UIDA* lines at the reproductive stage confirmed that *SAG12* was expressed in the root and especially localized in the stele (Figure 6). We noticed that despite higher *SAG12* expression in roots under LN than under HN, GUS staining was weaker in roots of LN plants. This was possibly due to the fact that the stronger protease activity measured under LN may have led to degradation of the b-glucuronidase enzyme, as previously shown in senescent leaf tissues by Noh and Amasino (1999a). Taken together, the expression of SAG12 in roots, and the increase in total protein content in the roots of *sag12* suggests that SAG12 is involved in the proteolysis associated with the root N remobilization, particularly when plants are facing N limitation.

This assumption was verified by measuring the total- and cysteine-protease activities in the roots of Col and *sag12* at the reproductive stage. While the depletion of SAG12 in *sag12* (SALK_124030) did not affect the total protease activity in roots, it decreased significantly the cysteine protease activity of the roots of plants cultivated under LN conditions.

Altogether, the results suggest that the SAG12 protease could plays a major role in the breakdown of root proteins when plants are facing N limitation and is required for efficient N remobilization to the seeds.

## Conclusion

For the first time, this work shows a clear expression of SAG12 in the stele of the roots. This expression pattern associated with results obtained in *sag12* (SALK_124030) suggest a role of SAG12 for N remobilization from roots to support seed production and seed N content under N limitation. This study open new ways for improving N use efficiency in crops. For example, in *Brassica napus*, the level of SAG12 root expression could have partly explained the contrasting N use efficiency highlighted by Girondé et al. (2015a,b) in different genotypes cultivated under N limiting conditions.

## Author Contributions

MJ, CM-D, PE, and JT conducted all the experiments, analyzed the data, and wrote the manuscript. PL performed N uptake capacity experiments and wrote the manuscript. DG performed microscopic analysis. MA and AM realized plasmid constructions, transformation and selection of transgenic lines.

## Conflict of Interest Statement

The authors declare that the research was conducted in the absence of any commercial or financial relationships that could be construed as a potential conflict of interest.
