# Supplementary material for: A New Role for SAG12 Cysteine Protease in Roots of Arabidopsis thaliana
Source: Front Plant Sci. 2019 Jan 11;9:1998. doi: 10.3389/fpls.2018.01998 (PMC6337903; doi:10.3389/fpls.2018.01998)
Supplement: Supplementary file 1 [file Image_1.pdf]

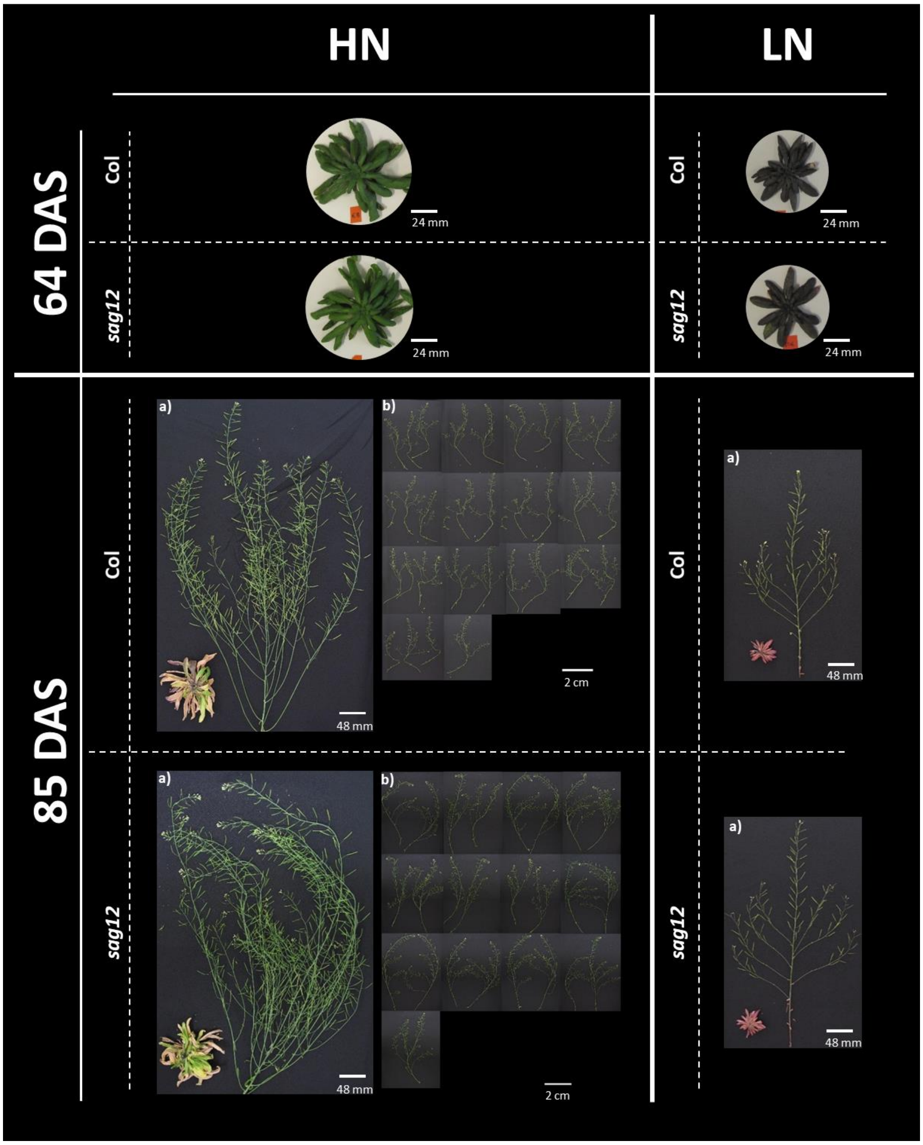

Figure S1: Phenotypes of CoL and *sag12* plants at 64 DAS and 85 DAS. Pictures of representative Col and *sag12* plants at the vegetative stage (64 DAS) and the reproductive stage (85 DAS) grown under high nitrogen (HN) or low nitrogen (LN) conditions. At the reproductive stage (85 DAS), plants were split into the rosette and the main stem (a) and for HN condition the secondary stems (b). For each picture, the scale is indicated by the white bar.

Figure S1 James et al.
